# A cross-language speech model for detection of Parkinson’s disease

**DOI:** 10.1007/s00702-024-02874-z

**Published:** 2024-12-30

**Authors:** Wee Shin Lim, Shu-I Chiu, Pei-Ling Peng, Jyh-Shing Roger Jang, Sol-Hee Lee, Chin-Hsien Lin, Han-Joon Kim

**Affiliations:** 1 Department of Computer Science and Information Engineering, National Taiwan University, Taipei, Taiwan; 2 Department of Computer Science, National Chengchi University, Taipei, Taiwan; 3 Department of Neurology, College of Medicine, National Taiwan University Hospital, National Taiwan University, Taipei, 100 Taiwan; 4 Department of Neurology, Seoul National University Hospital and Seoul National University College of Medicine, Seoul, Korea; 5 Colleague of Medicine, National Taiwan University, Taipei, Taiwan; 6 Department of Biomedical Engineering, National Taiwan University, Taipei, Taiwan; 7 Institute of Molecular Medicine, College of Medicine, National Taiwan University, Taipei, Taiwan

**Keywords:** Parkinson’s disease, Biomarkers, Speech, Face, Deep-learning model

## Abstract

Speech change is a biometric marker for Parkinson’s disease (PD). However, evaluating speech variability across diverse languages is challenging. We aimed to develop a cross-language algorithm differentiating between PD patients and healthy controls using a Taiwanese and Korean speech data set. We recruited 299 healthy controls and 347 patients with PD from Taiwan and Korea. Participants with PD underwent smartphone-based speech recordings during the “on” phase. Each Korean participant performed various speech texts, while the Taiwanese participant read a standardized, fixed-length article. Korean short-speech (≦15 syllables) and long-speech (> 15 syllables) recordings were combined with the Taiwanese speech dataset. The merged dataset was split into a training set (controls vs. early-stage PD) and a validation set (controls vs. advanced-stage PD) to evaluate the model's effectiveness in differentiating PD patients from controls across languages based on speech length. Numerous acoustic and linguistic speech features were extracted and combined with machine learning algorithms to distinguish PD patients from controls. The area under the receiver operating characteristic (AUROC) curve was calculated to assess diagnostic performance. Random forest and AdaBoost classifiers showed an AUROC 0.82 for distinguishing patients with early-stage PD from controls. In the validation cohort, the random forest algorithm maintained this value (0.90) for discriminating advanced-stage PD patients. The model showed superior performance in the combined language cohort (AUROC 0.90) than either the Korean (AUROC 0.87) or Taiwanese (AUROC 0.88) cohorts individually. However, with another merged speech data set of short-speech recordings < 25 characters, the diagnostic performance to identify early-stage PD patients from controls dropped to 0.72 and showed a further limited ability to discriminate advanced-stage patients. Leveraging multifaceted speech features, including both acoustic and linguistic characteristics, could aid in distinguishing PD patients from healthy individuals, even across different languages.

**Supplementary Information:**

The online version contains supplementary material available at 10.1007/s00702-024-02874-z.

## Introduction

With the advancement of technology and artificial intelligence, an increasing number of studies is being performed on digital biomarkers for early detection of disease or assistance in diagnosis. In Parkinson’s disease (PD), digital biomarkers are being developed for motor symptoms, cognition, mood, sleep, speech, and autonomic functions (Alfalahi et al. 2023). Among them, speech is one of the most studied biometric markers. Speech in patients with PD is characterized by breathy phonation, hoarseness, low speech volume, inaccurate articulation, and monotonous speech, which occurs in 70–90% of patients with PD at some point during the course of the disease and even in the early and prodromal stages (Ho et al. 1998; Jeancolas et al. 2022; Rusz et al. 2021).

Studies of speech algorithms to differentiate patients with PD from healthy controls (HCs) have been developed in more than 30 languages, and the results show accuracy ranging from 0.67 to 1.0 (Idrisoglu et al. 2023). This wide discrepancy stems from the difference in demographic characteristics, disease severity, recording task, and speech analysis methods. In addition, it is also likely that the unique features of each language, including variations in pronunciation (articulation and clarity) and phonetics (acoustic properties such as pitch, rhythm, and tonal structure), affect the performance of the algorithm used in each study.

This study focuses on speech analysis, which includes both acoustic features (e.g., pitch variability, intensity) and linguistic features (e.g., speech rate, articulation errors). Speech in patients with PD is often characterized by changes in both phonation and articulation, which can manifest as reduced fundamental frequency variability, slower speech rate, and higher pause percentage. The pattern of speech changes in Parkinson's disease (PD) has been studied in different language groups, including Mandarin, Japanese, and Thai (Idrisoglu et al. 2023; Favaro et al. 2023; Laganas et al. 2021). However, a cross-language algorithm that can differentiate PD from controls independently of language has not been thoroughly explored across diverse linguistic settings. To date, few studies have investigated whether a single algorithm can differentiate patients with PD from HCs in multiple languages (Favaro et al. 2023; Laganas et al. 2021). In one study on a mixed population of PD patients speaking English, Greek, German, and Portuguese, the language-unaware classification process showed a diagnostic accuracy of 0.7 in the differentiation of speech from patients with PD from HCs, which was lower than the language-aware method for each language (Laganas et al. 2021). Another study analyzed acoustic features of American English, Italian, Castilian Spanish, Colombian Spanish, German, and Czech speech to differentiate PD from HCs independent of language (Favaro et al. 2023). Various models were used, and the accuracy ranged from 0.56 to 0.92. All the languages included in the above studies are Indo-European languages. Recent machine learning studies focusing on Asian languages, including Korean (Jeong et al. 2024), Thai (Bhidayasiri et al. 2024), Japanese (Yokoi et al. 2023), and Mandarin (Zhang et al. 2023) have demonstrated promising results for speech-based diagnostics, further highlighting the potential of cross-lingual approaches. However, to our knowledge, no study has established an algorithm for identifying PD in a multilingual setting in Asia, particularly between languages belonging to different language families that are phonetically distinct. In this study, we developed a cross-language algorithm to differentiate between patients with PD and HCs using Taiwanese speech, which belongs to the Sino-Tibetan family, and Korean speech, which belongs to the Altaic family. Korean and Taiwanese Mandarin differ significantly in their phonetics and linguistic structure. Korean, an Altaic language, has a syllable-timed rhythm and uses Hangul characters, where each syllable corresponds to one character. In contrast, Taiwanese Mandarin, a Sino-Tibetan language, employs logograms, with intonation playing a critical role in meaning. Despite these differences, both languages share speech characteristics relevant to PD, such as changes in pitch and pauses, which could be adopted for cross-lingual studies. Herein, we developed a cross-language algorithm differentiating between patients with PD and HCs using Taiwanese speech, which belongs to the Sino-Tibetan family, and Korean speech, which belongs to the Altaic family.

## Materials and methods

### Study participants

A total of 646 participants, including 299 healthy controls and 347 patients with PD, were recruited from movement disorder clinics of Seoul National University Hospital (125 controls and 161 patients with PD) and National Taiwan University Hospital (174 controls and 186 patients with PD). PD was diagnosed according to the United Kingdom PD Society Brain Bank Clinical Diagnostic Criteria (Hughes et al. 1992). The controls were neurologically unaffected participants who were spouses or friends accompanying the patients with PD. We excluded participants who were illiterate, as well as those with hearing impairments or other non-neurological disorders that could affect the vocal cords. All participants underwent otolaryngologic evaluations. All Korean and Taiwanese participants had received at least 9 years of education, which is the compulsory education requirement in these regions. Among PD patients, early-stage PD was defined as Hoehn–Yahr stage < 2.5 and advanced-stage PD was defined as a Hoehn–Yahr stage ≥ 2.5. All participants provided written informed consent, and the institutional ethics boards of Seoul National University Hospital and National Taiwan University Hospital approved the study.

### Speech datasets

#### Multidisciplinary approach to dataset and task selection

The selection of speech datasets and tasks was guided by a multidisciplinary team comprising movement disorder specialists, machine learning specialists, and a speech analysis expert. This collaborative approach ensured that the tasks were linguistically and clinically appropriate for identifying PD-related speech changes. Task selection, such as reading speech in Korean and Mandarin, was informed by language-specific methodologies, ensuring the inclusion of tailored tasks critical for capturing distinct linguistic features relevant to PD and the respective languages. Different stimuli were used for Korean and Taiwanese datasets because the two languages belong to different language families (Altaic and Sino-Tibetan, respectively) and exhibit distinct phonetic and linguistic characteristics. Using stimuli that align with each language's natural phonetic structure ensures robust analysis of PD-related speech changes while maintaining linguistic validity.

#### Korean dataset

**Fig. 1:**
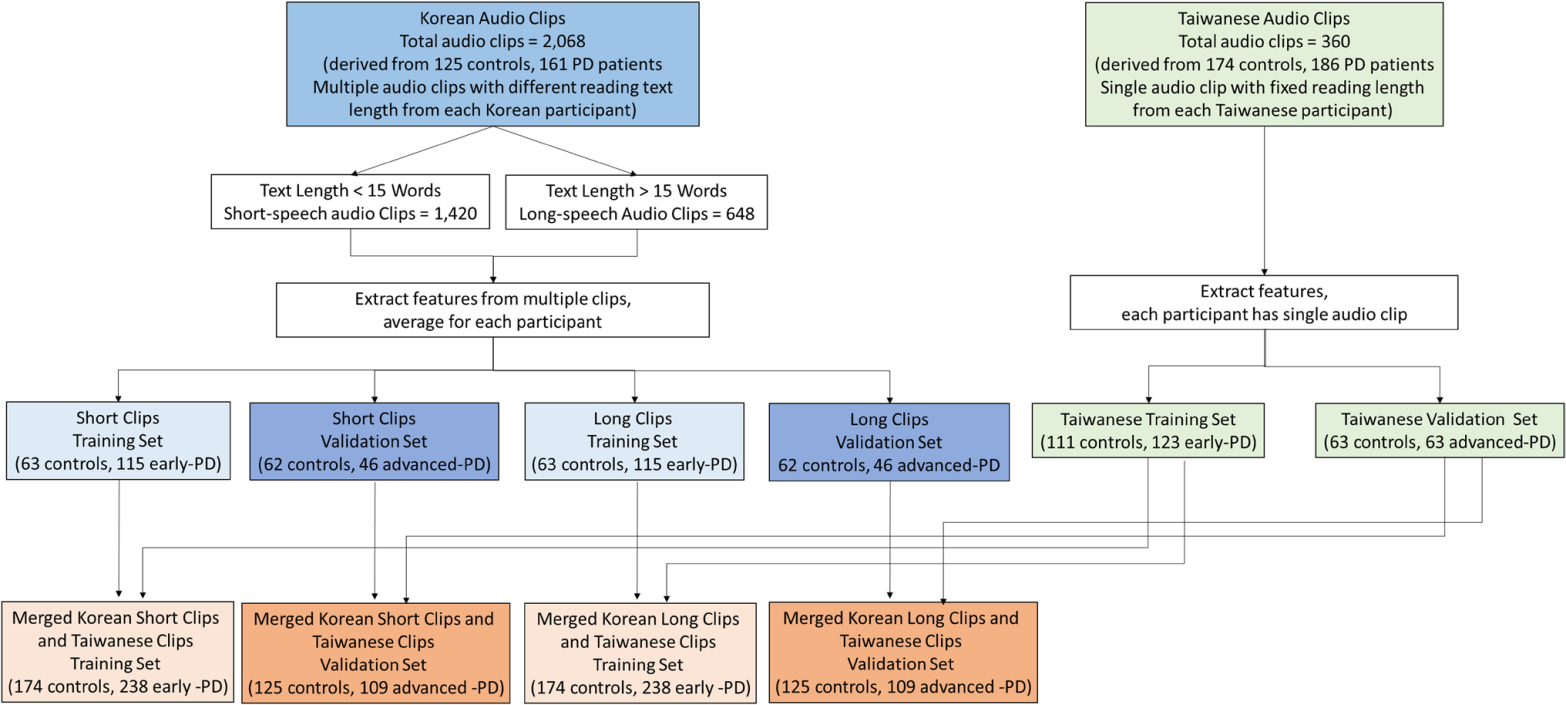
Study design flowchart with two cohorts. In the Korean cohort, participants performed various speech texts, while the Taiwanese participant read a standardized, fixed-length article. Korean short-speech (≦15 syllables) and long-speech (> 15 syllables) recordings were combined with the Taiwanese speech dataset. The merged dataset was then divided into a training set (controls vs. early-stage PD) and a validation set (controls vs. advanced-stage PD) to evaluate the effectiveness of a speech length-based model in distinguishing PD patients from unaffected controls across languages

The Korean dataset included both short and long sentences designed to capture a wide range of phonetic and linguistic contexts. These stimuli were carefully selected to incorporate variations in vowels, consonants, and sentence structures reflective of the Korean language. Additionally, the sentences were chosen to capture natural prosody and syntactic diversity. The Korean speech recordings were gathered from 291 Korean participants, including 125 control individuals and 161 patients with PD. Each participant was asked to perform 12 distinct Korean speech texts (detailed description were shown in Supplementary Data) or speech tasks, including sustained vowel phonation (Naranjo et al. 2017), syllable repetition tasks (Skodda et al. 2013), sentence repetition tasks (Bandini et al. 2016), and reading tasks (Galaz et al. 2016), on the same day during the “on” phase of medication, resulting in a total of 2,068 Korean audio clips. Among the 2,068 Korean audio clips, 1420 clips contained reading text lengths of less than 40 characters (short-speech recordings) and 648 clips contained reading text length exceeding 40 characters (long-speech recordings) (Fig. 1 and Supplementary Table 1). Audio features were extracted from multiple clips, and the average values were calculated for each participant.

#### Taiwanese dataset

The Taiwanese dataset consisted of a single, standardized passage that represents key linguistic features of Taiwanese Mandarin, such as tonal variations and frequent phonemes characteristic of the language. This passage was selected for its linguistic richness, incorporating all major tones and a diverse set of vowels and consonants. It facilitates the analysis of tonal dynamics, which are particularly relevant to Taiwanese Mandarin and are influenced by PD. Taiwanese speech recordings were derived from a published speech dataset of 360 Taiwanese participants, including 174 controls and 186 patients with PD, who were requested to read a standardized article with a fixed-length text consisting of 500 characters (Lim et al. 2022). The 360 Taiwanese audio clips contained standardized long text readings derived from each participant. Among the Taiwanese speech dataset of patients with PD, only speech files recorded during the “on” medication phase were used in the current study.

#### Recording process

All recordings were conducted in a quiet indoor clinic environment to minimize background noise. While the environment was not a professional soundproof studio, care was taken to minimize external disturbances, with the recording setup kept consistent across participants. Participants were instructed to speak naturally and maintain a steady tone and volume during the tasks. Trained research assistants supervised all recordings, and the same model of smartphone was used for consistency in each dataset. The smartphone microphone was positioned at a consistent distance of 5–35 cm from the participant’s mouth, and this setup was verified before each recording session. Speech data were initially captured in linear PCM format (.wav) with a sampling rate of 44.1 kHz and a 24-bit sample size, then down sampled to 44.1 kHz and 16-bit for uniform processing. These recordings were categorized according to the length of their speech content. Long-speech recordings were characterized by a lack of repetitive words and a minimum of 40 Hangul characters, whereas short-speech recordings were defined by a length of fewer than 40 characters. In Korean, as in Taiwanese, one character represents one syllable.

#### Dataset merging and model training

To evaluate the sensitivity of a cross-lingual speech model in distinguishing patients with Parkinson’s disease (PD) from healthy controls, we combined the Korean and Taiwanese speech datasets. Considering the uneven number of speech datasets derived from each participant in the two cohorts, they were merged in two distinct ways First, the Korean long-speech recordings (≥ 40 Hangul characters) were combined with the Taiwanese dataset (500 characters). Second, the Korean short-speech recordings (< 40 Hangul characters) were merged with the same Taiwanese dataset. Furthermore, the merged datasets were then split into training and testing (validation) sets based on disease stage. The training set included early-stage PD patients (Hoehn–Yahr stage ≤ 2) and healthy controls, while the testing/validation set comprised advanced-stage PD patients (Hoehn–Yahr stage > 2) and an independent group of controls (Fig. 1). The testing data included participants from both cohorts to assess the model's cross-lingual generalizability. This approach provided a unique opportunity to evaluate the model’s effectiveness in distinguishing PD patients from controls across varying speech lengths and linguistic contexts (Fig. 1). By dividing the speech recordings into training (early-stage PD patients and controls) and validation (advanced-stage PD patients and another control group) datasets for each merged configuration, we ensured robust testing of the model’s performance.

All PD patients were receiving levodopa therapy, and the speech was recorded during the “on” phase, such that the motor function and speech of patients with early-stage PD would be similar to those observed in healthy older individuals. Therefore, patients with early-stage PD should be more difficult to differentiate from controls than patients with advanced-stage PD. For this reason, we trained the model with speech features derived from patients with early-stage PD to differentiate them from healthy controls. We reasoned that a model that could discriminate patients with early-stage PD from healthy controls might show optimal diagnostic performance for identifying drug-naïve patients with PD or advanced-stage PD among healthy older individuals.

### Speech feature selection

Several speech features were used to distinguish patients with PD from healthy controls. Because patients with PD present with hypovolemic and monotonous speech (Lim et al. 2022), speech volume and fundamental frequency (pitch) features were adopted as features that could discriminate patients with PD from controls. Volume represents the vocal intensity of an audio signal, which correlates with the amplitude of the signal. Therefore, we adopted volume variance, pause percentage, fundamental frequency variability, and average fundamental frequency as speech features in our model. Vocal intensity variance was calculated by analyzing variations in the volume of audio frames across the speech sample, while fundamental frequency variance was extracted frame-by-frame using Python’s pysptk library. Pause percentage was derived as the proportion of silent frames to total recording length, with silent frames identified based on a dynamically set threshold. Average fundamental frequency was computed as the mean F0 across voiced frames after pitch tracking. All stimuli were fully analyzed for consistency, using Python-based signal processing tools. While other vocal parameters (e.g., jitter, shimmer, and HNR) are affected by PD, cross-lingual applicability challenges led to their exclusion. The inclusion of speech-related measures aimed to complement acoustic features by capturing linguistic and articulatory characteristics of PD.

In addition to volume and fundamental frequency, intonation, pronunciation, and syllable length vary between the two languages; therefore, we used the ground-truth speech text provided to the participants at recording and the Google Speech-to-Text API, which is versatile and can be applied to all supported languages (Google 2023a, b). We utilized the Google Speech-to-Text API for transcribing the Korean speech dataset into Korean text and Taiwanese Mandarin speech to traditional Mandarin text. After transcription, we measured the following features. (1) Speech Rate: Calculated as the ratio of reading duration to text character length, this measure assesses spoken language efficiency by focusing on the pace of speech, not the overall clip length. A lower ratio indicates faster speech, allowing for accurate comparison across both short and long audio clips. (2) Speech-to-Text Google API Confidence Score: This score reflects the API’s confidence in the accuracy of its transcription. A higher confidence score suggests that the transcribed text is a more accurate representation of the original speech, which is crucial for assessing transcription reliability. (3) Speech-to-Text Word Error Rate (WER): The WER is a standard metric for evaluating speech recognition accuracy. This involves comparing the API-generated transcription with a ground-truth text to quantify discrepancies. A lower WER signifies higher accuracy, indicating the system's effectiveness in converting spoken language into written form. This metric is particularly valuable for evaluating the effects of factors such as background noise, accents, and linguistic variations on transcription accuracy.

### Machine learning algorithms and analyses

We used sequential forward feature selection with base classifiers such as Random Forest (Breiman 2001), Support Vector Machine (SVM) (Pisner and Schnyer 2020), and AdaBoost (Freund and Schapire 1997) to train our model. The source code for all classifiers is available in the python science-kit learning library (Buitinck et al. 2013). We implemented the leave-one-out cross-validation (LOOCV) method to reduce both bias and variance in the machine learning models by providing an objective estimate of the model's performance on new data. In LOOCV, the model is trained on all data points except one, and this process is repeated for each data point, ensuring that every observation is used for both training and validation. We compared the performances of these training classifiers based on several key performance metrics. These metrics included the accuracy, precision, recall, F1-score, and area under the receiver operating characteristic curve (AUROC) for binary classification. This comprehensive evaluation approach allowed us to assess the effectiveness of each classifier in the context of our study, ensuring a robust and reliable machine learning model.

### Statistical analysis

Continuous variables are expressed as mean ± standard deviation. For variables where appropriate, we have also included the median and interquartile range in addition to the mean. Categorical variables are expressed as number and percentage. We tested the homogeneity of variances using Levene’s tests. Variables were compared using two-tailed *t* tests or analysis of variance (ANOVA) when normally distributed, or with the non-parametric t-test when assumptions of normality or homoscedasticity were violated. The diagnostic performance of the models is expressed as the AUROC and 95% confidence interval (95% CI). All statistical analyses were performed using SAS (version 9.4; Cary, NC, USA) and GraphPad Prism (version 9.0.0; San Diego, California, USA). P values < 0 0.05 were considered statistically significant.

## Results

The demographic data of all enrolled participants from the Korean and Taiwanese cohorts are shown in Table 1. In both cohorts, patients with PD were older than controls. In addition, the PD group had a higher percentage of men than the control group.

**Table 1:** Clinical characteristics and voice features of all study participants derived from Korean and Taiwanese cohorts

	Korean cohort						Taiwanese cohort					
	Training dataset			Validation dataset			Training dataset			Validation dataset		
	Controls n = 63	Early PD n = 115	*P* value	Controls n = 62	Advanced PD n = 46	*P* value	Controls n = 111	Early PD n = 123	*P* value	Controls n = 63	Advanced PD n = 63	*P* value
Male sex, N (%)	23 (36.5)	64 (55.7)	< 0.01**	18 (29.0)	20 (43.5)	< 0.01**	43 (38.7)	62 (50.4)	0.05	28 (44.4)	42 (66.7)	0.01
Current age, year	60.1 ± 15.7	65.7 ± 9.8	< 0.01**	44.0 ± 17.5	71.2 ± 7.2	< 0.01**	68.8 ± 9.1	66.4 ± 9.7	0.05	69.0 ± 8.6	71.2 ± 7.5	0.13
H-Y stage (on)	N.A	1.7 ± 0.4		N.A	3.1 ± 0.6		N.A	1.8 ± 0.3		N.A	3.7 ± 0.5	
Pause percentage mean ± SD (median ± IQR)	15.2 ± 8.2 (14.4 ± 9.6)	15.7 ± 8.3 (15.0 ± 9.2)	0.25	12.4 ± 17.1 (11.9 ± 8.8)	17.1 ± 9.9 (17.0 ± 12.3)	< 0.01**	13.5 ± 7.2 (12.5 ± 8.7)	21.7 ± 10.4 (20.7 ± 12.1)	< 0.01**	11.7 ± 6.4 (11.1 ± 9.1)	29.8 ± 12.1 (28.4 ± 18.0)	< 0.01**
Volume variance mean ± SD (median ± IQR)	– 2.4 ± 12.7 (– 1.5 ± 14.8)	0.5 ± 12.4 (0.5 ± 14.0)	< 0.01**	– 3.1 ± 9.9 (– 1.2 ± 14.8)	– 0.5 ± 12.7 (0.52 ± 12.7)	< 0.01**	– 1.9 ± 11.2 (– 0.8 ± 13.8)	1.1 ± 10.8 (1.2 ± 12.5)	0.03	– 1.4 ± 9.6 (– 0.7 ± 10.5)	2.5 ± 10.2 (3.1 ± 12.1)	< 0.01**
Pitch variance mean ± SD (median ± IQR)	13.1 ± 4.8 (12.5 ± 5.7)	11.3 ± 4.1 (11.5 ± 5.4)	< 0.01**	13.8 ± 4.3 (13.6 ± 5.5)	9.9 ± 3.9 (10.2 ± 5.7)	< 0.01**	16.7 ± 4.2 (16.7 ± 5.2)	13.1 ± 5.0 (12.9 ± 4.9)	< 0.01**	16.6 ± 4.7 (15.9 ± 6.8)	12.2 ± 4.3 (11.6 ± 4.0)	< 0.01**
Average pitch mean ± SD (median ± IQR)	185.8 ± 29.5 (192.7 ± 51.6)	176.0 ± 32.1 (184.5 ± 56.8)	< 0.01**	188.2 ± 31.7 (197.2 ± 22.8)	171.6 ± 31.8 (177.4 ± 44.7)	< 0.01**	156.5 ± 31.6 (158.0 ± 42.1)	152.9 ± 32.7 (160.1 ± 52.0)	0.39	152.8 ± 29.3 (159.0 ± 46.1)	151.1 ± 30.2 (149.0 ± 51.5)	0.74
Speech rate	0.2 ± 0.1	0.3 ± 0.1	< 0.01**	0.1 ± 0.1	0.2 ± 0.1	< 0.01**	0.2 ± 0.1	0.3 ± 0.2	< 0.01**	0.3 ± 0.1	0.4 ± 0.2	< 0.01**
Word error rate mean ± SD (median ± IQR)	0.6 ± 0.5 (0.4 ± 0.6)	0.5 ± 0.5 (0.5 ± 0.5)	0.13	0.5 ± 0.4 (0.4 ± 0.6)	0.6 ± 0.5 (0.5 ± 0.5)	< 0.01**	0.5 ± 0.2 (0.6 ± 0.2)	0.7 ± 0.2 (0.7 ± 0.2)	< 0.01**	0.6 ± 0.1 (0.6 ± 0.2)	0.8 ± 0.2 (0.8 ± 0.2)	< 0.01**
API confidence score mean ± SD (median ± IQR)	0.9 ± 0.1 (0.9 ± 0.1)	0.8 ± 0.2 (0.8 ± 0.1)	0.02*	0.9 ± 0.1 (0.9 ± 0.1)	0.8 ± 0.1 (0.9 ± 0.1)	< 0.01**	0.8 ± 0.2 (0.9 ± 0.3)	0.7 ± 0.2 (0.6 ± 0.4)	< 0.01**	0.8 ± 0.1 (0.9 ± 0.1)	0.6 ± 0.1 (0.6 ± 0.4)	< 0.01**

*SD* standard deviation, *IQR* interquartile range

In the Taiwanese cohort, in which reading a text was used as the source of speech data, we observed that all the speech-related features were significantly different between PD patients and controls, even those with early-stage PD during the “on” phase (Table 1). Patients with PD took longer to read the article, paused more during reading, had reduced fundamental frequency and volume variability, slower speech rate, higher word error rate, and lower API confidence scores than control participants. Similar patterns of speech features were observed in the Korean cohort. Although the Korean speech dataset contained versatile speech recordings from each participant, ranging from a single vowel sounds to reading text, the speech features of pause percentage and word error rate were comparable between patients with early-stage PD and controls. Furthermore, the API confidence score was only slightly different between patients with early-stage PD and ethnicity-matched controls.

**Fig. 2:**
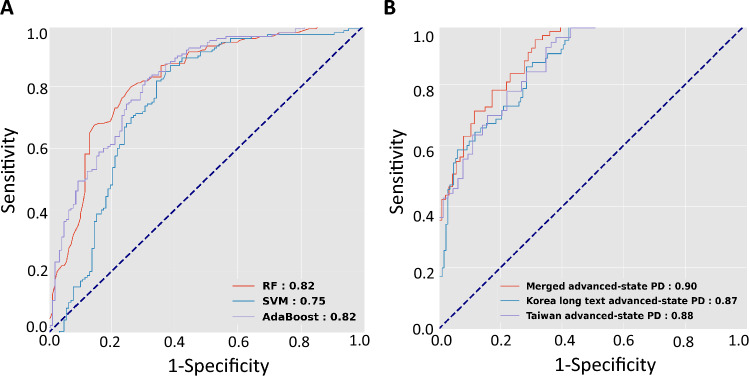
Receiver operating characteristic curves calculated with three deep-learning classifier models using long-text speech data. **A** The models were constructed using a training speech dataset sourced from early-stage PD patients of mixed ethnicity during the "on" phase and healthy controls. **B** The established model, employing a random forest classifier, was subsequently evaluated for its capacity to distinguish between patients with advanced PD in the "on" phase and an independent control group from a combined language cohort and populations exclusively utilizing either Korean or Taiwanese languages

Therefore, considering the heterogeneous recording length and speech tasks in the Korean speech dataset, we combined long Korean speech recordings with a minimum of 40 Hangul characters with the Taiwanese speech datasets. The clinical characteristics and speech features of the merged datasets are listed in Table 2. The training speech dataset was derived from patients with early-stage PD during the “on” phase and mixed-ethnicity controls (Table 2). In this merged training dataset, we integrated all speech features, including volume parameters and fundamental frequency characteristics, and API-related features, including speech rate, word error rate, and confidence score, combined with the basic characteristics of age and sex as an integrated cross-language model. We used a sequential forward selection method to select the best features for each classifier. The ROC analyses calculated with the random forest and AdaBoost classifiers provided the optimal diagnostic values of 0.82, for distinguishing patients with early-stage PD during the “on” phase from controls (Fig. 2A). Given the satisfactory performance of this cross-language model using a long-speech dataset, we then assessed whether this model might also be able to distinguish patients with advanced-staged PD during the “on” phase from a mixed language population. We found that the random forest classifier achieved high diagnostic performance, with an AUROC of 0.90, in identifying PD patients from set of mixed-ethnicity controls (Fig. 2B). Furthermore, under this classifier, the performance of the established model was better in the merged language cohort than either Korean or Taiwanese language datasets alone (Fig. 2B).

**Table 2:** Clinical characteristics and voice features of a merged cross-language dataset with long Korean speech recording

	Training dataset			Validation dataset		
	Controls, n = 174	Early PD, n = 238	*P* value	Controls, n = 125	Advanced PD, n = 109	*P* value
Male sex, N (%)	43 (32.1)	96 (60.0)	< 0.01**	24 (27.9)	46 (63.0)	< 0.01**
Current age, years	56.3 ± 18.6	65.6 ± 10.2	< 0.01**	44.9 ± 17.9	71.3 ± 5.9	< 0.01**
H-Y stage (on)	N.A	1.8 ± 0.7		N.A	3.3 ± 0.8	
Pause percentage mean ± SD (median ± IQR)	10.1 ± 6.6 (12.4 ± 8.1)	16.3 ± 8.4 (17.1 ± 11.0)	< 0.01**	9.7 ± 5.8 (9.8 ± 8.6)	20.5 ± 12.7 (26.9 ± 18.4)	< 0.01**
Volume variance mean ± SD (median ± IQR)	– 6.2 ± 11.5 (– 3.5 ± 14.0)	– 3.1 ± 10.2 (0.05 ± 11.9)	< 0.01**	– 6.4 ± 10.3 (– 2.7 ± 12.0)	– 0.8 ± 9.6 (1.9 ± 13.2)	< 0.01**
Pitch variance mean ± SD (median ± IQR)	15.3 ± 5.2 (16.1 ± 5.6)	11.6 ± 4.2 (12.4 ± 4.9)	< 0.01**	14.4 ± 4.0 (15.4 ± 5.6)	10.6 ± 4.2 (11.2 ± 4.0)	< 0.01**
Average pitch mean ± SD (median ± IQR)	172.4 ± 31.9 (162.0 ± 52.4)	165.0 ± 34.5 (161.5 ± 52.0)	< 0.01**	179.9 ± 36.2 (167.5 ± 63.5)	158.3 ± 33.5 (151.0 ± 51.1)	< 0.01**
Word error rate mean ± SD (median ± IQR)	0.4 ± 0.2 (0.5 ± 0.3)	0.5 ± 0.2 (0.6 ± 0.3)	< 0.01**	0.4 ± 0.2 (0.5 ± 0.3)	0.6 ± 0.3 (0.8 ± 0.3)	< 0.01**
API confidence score mean ± SD (median ± IQR)	0.8 ± 0.1 (0.9 ± 0.1)	0.8 ± 0.2 (0.8 ± 0.4)	< 0.01**	0.9 ± 0.1 (0.9 ± 0.1)	0.7 ± 0.2 (0.7 ± 0.4)	< 0.01**
Speech rate	0.2 ± 0.1	0.2 ± 0.1	0.06	0.2 ± 0.1	0.3 ± 0.2	< 0.01**

*SD* standard deviation, *IQR* interquartile range

**Fig. 3:**
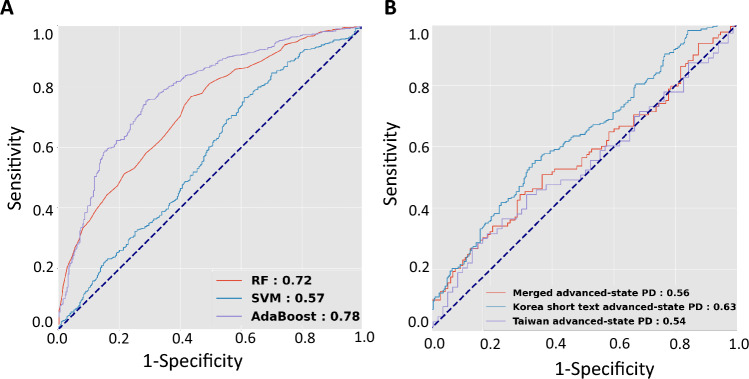
Receiver operating characteristic curves calculated with three deep-learning classifier models using short-text speech data. **A** The models were constructed using a training speech dataset sourced from early-stage PD patients of mixed ethnicity during the "on" phase and healthy controls. **B** The established model, employing a random forest classifier, was subsequently evaluated for its capacity to distinguish between patients with advanced PD in the "on" phase and an independent control group from a combined language cohort and populations exclusively utilizing either Korean or Taiwanese languages

We then examined whether speech length affects the diagnostic accuracy of identifying patients with PD from controls. Another merged speech dataset that included Korean short-speech recordings with a length of less than 25 characters and the Taiwanese speech datasets was prepared. We found that the diagnostic performance was marginal for the training dataset using speech files from patients with early-stage PD during the “on” phase and controls. The AdaBoost and random forest classifiers provided the highest performance with AUROCs of 0.78 and 0.72, respectively (Fig. 3A). However, while applying the merged model built from the short-speech dataset to the validation cohort of mixed-language patients with advanced-stage PD and another subset of controls, all the classifiers showed limited diagnostic performance in discriminating advanced-stage PD patients from normal subjects (Fig. 3B). The random forest classifier only provided a diagnostic accuracy with an AUROC score of 0.56 in the validation dataset.

Furthermore, to assess the impact of sex and age on model performance, we conducted subgroup analyses by first developing separate models for each biological sex. Additionally, we restricted the analysis to participants aged 40 and above in both the Taiwanese and Korean datasets. These results demonstrated that, among females, the integrated voice model using the long-speech dataset achieved high performance with an AUROC of 0.95 (Supplementary Fig. 1A). Among males, the model also performed satisfactorily, with an AUROC of 0.88 (Supplementary Fig. 1B). In the cohort of participants aged 40 and older in the merged dataset, the integrated voice model achieved an AUROC of 0.83 (Supplementary Fig. 1C). Taken together, these observations demonstrate that a cross-language model using multifaceted speech features with a suitable and long length of speech recordings could assist the identification of PD patients from controls. The short speech dataset, including simple vowel sounds or repetitive wording, may not provide enough information to differentiate patients with PD from controls.

## Discussion

In this study, we established a speech model that incorporates a variety of acoustic and linguistic features that can satisfactorily discriminate patients with PD from control participants across two different languages. The model demonstrated an optimal diagnostic performance for distinguishing between patients with early-stage PD and controls using speech data derived from a long length of speech recording, whereas the diagnostic performance of a model using short speech recordings or vowel sounds alone was not as effective. Furthermore, the model using the random forest classifier was validated with another independent mixed-language cohort that comprised patients with advanced-stage PD and another group of controls. Of note, the performance of the established model is superior in the merged language cohort compared to either the Korean or Taiwanese language cohorts individually.

Our findings suggest that models trained on datasets enriched with multiple languages could provide satisfactory diagnostic capabilities in differentiating patients with PD from controls. In contrast to most of the previous studies that mainly analyzed the performance of these models considering one language at a time and a unique speech task (e.g., reading task, diadokinetic task) or features encoding one type of information only (e.g., acoustic), our model used integrated features, including speech volume and speech pitch, and adopted the all-language amenable Speech-to-Text Google API derived speech rate, Speech-to-Text Google API Confidence Score, and WER. These speech features, therefore, combined the integration of acoustic and linguistic approaches that could compensate for the variations of intonation, pronunciation, and syllable length among different languages. The speech-to-text features, such as WER, Confidence Score, and Speech Rate, are robust across languages and do not require major language-specific adjustments. This makes them more suitable for cross-lingual analysis compared to formant analysis, depends heavily on vowel structures, which vary significantly between languages (Barrientos and Cataldo 2023), such as Taiwanese Mandarin and Korean in this study. These variations necessitate extensive language-specific tuning, which would complicate cross-lingual comparisons. Furthermore, formant analysis is often biased by strong harmonics in the signal, shifting measurements away from true vocal tract resonances. (Whalen et al. 2022). This issue becomes more pronounced in noisy or uncontrolled environments, which are inevitable in real-world data collection. Additionally, formant analysis requires high-quality recordings and advanced methods to reduce errors, which are often not fully automated (Zaltz et al. 2020). These requirements limit its scalability and practicality for large datasets or remote applications. In contrast, STT features can be derived using existing technologies, such as the Google Speech-to-Text API, and are well-suited for large-scale and real-world applications, including remote monitoring. By focusing on STT features, our study ensures broader applicability, particularly for a cross-language dataset, where formant analysis poses significant challenges.

One recent cross-lingual study integrating both acoustic and linguistic features of six speech datasets from a relatively limited number of mixed European and American participants obtained a variable diagnostic accuracy based on mono-lingual (85%), multi-lingual (88%), and cross-lingual experiments (79%) (Favaro et al. 2023). A cross-language experiment in three different languages, including Spanish, German and Czech, using four speech tasks comprising isolated vowel words, rapid repetition of the syllables /pa/-/ta/-/ka/, sentences, and reading texts, showed accuracies ranging from 85 to 95%, with text reading showing the highest performance (Orozco-Arroyave et al. 2016). Another study using a running speech dataset from phone calls in a mixed population of PD patients speaking English, Greek, German, and Portuguese containing dozens of participants in each language subset demonstrated a diagnostic accuracy of 0.7 AUROC in differentiating patients with PD from controls in the language-unaware analysis (Laganas et al. 2021). Most of the abovementioned published studies enrolled patients with PD as a whole without further subdividing them into early-stage or advanced-stage PD, which may cause a heterogeneous performance of the established speech model. The various factors influencing the ability to distinguish between patients with PD and controls may stem from several sources. These include the sample size utilized in the study, whether participants were on or off medication during the speech recording, and the specific speech tasks employed. Our cross-lingual enhancement model based on different speech features obtained higher AUROC scores for the merged datasets than those with single-language data. Further large-scale studies including more languages, especially western and other Asian languages, are needed to confirm our findings.

Our results also demonstrated that performance of long-length speech tasks is better than that of short-length readings or single vowel sound articulations. The comparison between Figs. 1B and 2B reveals intriguing insights into the impact of text length on the diagnostic performance of machine learning algorithms for PD. While Fig. 1B demonstrated strong validation results with a Random Forest classifier on long-text datasets, achieving an AUROC of 0.90 for the merged Korean long text and Taiwanese advanced-state PD cohort, the outcomes with short-text data in Fig. 2B showed a notable reduction in diagnostic performance with an AUROC of 0.56. Specifically, the AUROC for the merged advanced-stage PD cohort in Fig. 2B, using short-text data, is 0.56, which is 34% lower than the AUROC obtained with long-text data shown in Fig. 1B. This substantial difference suggests that reading short texts may not provide sufficient linguistic features for the algorithms to accurately discriminate between patients with early-stage PD and control subjects, especially when the disease's linguistic markers are subtle and less pronounced. In support of our findings, one recent study comparing performance between long text reading (more than 7 sentences) and single vowel sounds to identify patients with different types of dysphonia from healthy controls showed a marked diagnostic accuracy superiority of long text reading (78.12%) compared with single vowel sounds alone (50.92%) (Wang et al. 2022). This finding reinforces the hypothesis that longer texts capture richer linguistic features that are crucial for the accurate identification of PD. Moreover, it indicates that while combining datasets in multiple languages can enhance model robustness, the length of the input text remains a critical factor for maintaining high diagnostic accuracy.

By integrating both acoustic and linguistic speech features, our study provides a comprehensive framework for analyzing PD-related speech changes. This approach overcomes the limitations of sustained phonation by addressing a range of speech characteristics, including prosody, articulation, and fluency. Such diversity enhances diagnostic sensitivity and facilitates cross-lingual applicability. Looking ahead, leveraging smartphone technology and cloud-based analysis could enable remote monitoring of PD symptoms. Incorporating speech analysis with wearable devices could offer a multimodal diagnostic solution, while personalization and cross-cultural validation could improve model robustness and clinical impact. Researchers and physicians could analyze these biometric features remotely to identify patients who may have PD, without the need for an in-person interview. These directions highlight the potential for translating speech-based diagnostics into scalable, practical solutions for real-world healthcare settings, particularly benefiting patients in remote or underdeveloped areas without access to movement disorder specialists.

Our study, while pioneering in its approach to cross-lingual and text-length variations in assisting PD identification, has the following limitations. One important constraint is the potential imbalance in the representation of languages within the datasets, which may have affected the model's learning process. Additionally, the study did not account for dialectal variations within the Korean and Taiwanese populations, which could have substantive implications for linguistic biomarkers of PD. On the other hand, we accounted for differences between tonal and non-tonal languages by applying statistical normalization techniques to standardize fundamental frequency (F0) features across Taiwanese Mandarin and Korean. However, the unique tonal patterns in Taiwanese Mandarin may introduce linguistic variations that are not present in Korean, highlighting a limitation that warrants further investigation in future studies. Furthermore, the female percentage was higher in the control group than in the PD group. Speech formation differs between sexes due to anatomical and physiological differences in the phonatory system. These differences contribute to distinct acoustic parameters, such as jitter (more altered in men) and fundamental frequency (higher in women due to greater number of vocal fold vibratory cycles) (Lovato et al. 2016). Although we performed subgroup analyses demonstrating that the integrated model performed well in both males and females, as well as in participants aged 40 and older, further studies are needed that include more participants, use different languages, and ensure a balanced age and sex distribution in both groups to confirm our findings. In addition to acoustic features, we also incorporated linguistic characteristics in our model, which minimized the effects of sex in our model’s diagnostic performance to identify patients with PD from controls. Another limitation of this study is the lack of information regarding the medication status of the Korean participants. All recordings were conducted during the "ON" phase to ensure that motor symptoms were controlled during speech recording. However, there may be a potential impact of anti-parkinsonism medications on the speech features of participants. Previous studies have suggested that the use of levodopa can improve vocal parameters such as fundamental frequency and jitter; however, speech intensity remains reduced in both the "ON" and "OFF" states of therapy (Pinho et al. 2018). A future study including drug-naïve PD patients, free from the effects of anti-parkinsonism medications, is needed to confirm our findings. Additionally, while this study focused on quantitative features, qualitative aspects like timbre and speech clarity, which may vary between early- and late-stage PD, should be explored in future research to provide a more holistic understanding of PD-related changes.

In conclusion, our findings indicate that leveraging multifaceted speech features that encompass both acoustic and linguistic characteristics aid in distinguishing patients with PD from healthy individuals, even across different languages.

## Supplementary Information

Below is the link to the electronic supplementary material.

Supplementary file1 (PPTX 441 kb)

Supplementary file2 (DOCX 2639 kb)

Supplementary file3 (DOCX 20 kb)

## Data Availability

Data available from the corresponding author on reasonable request.
