# Supplementary material for: A cross-language speech model for detection of Parkinson’s disease
Source: J Neural Transm (Vienna). 2024 Dec 30;132(4):579–90. doi: 10.1007/s00702-024-02874-z (PMC11909049; doi:10.1007/s00702-024-02874-z)
Supplement: Supplementary file 1 — Supplementary file1 (PPTX 441 kb) [file 702_2024_2874_MOESM1_ESM.pptx]

## Slide 1
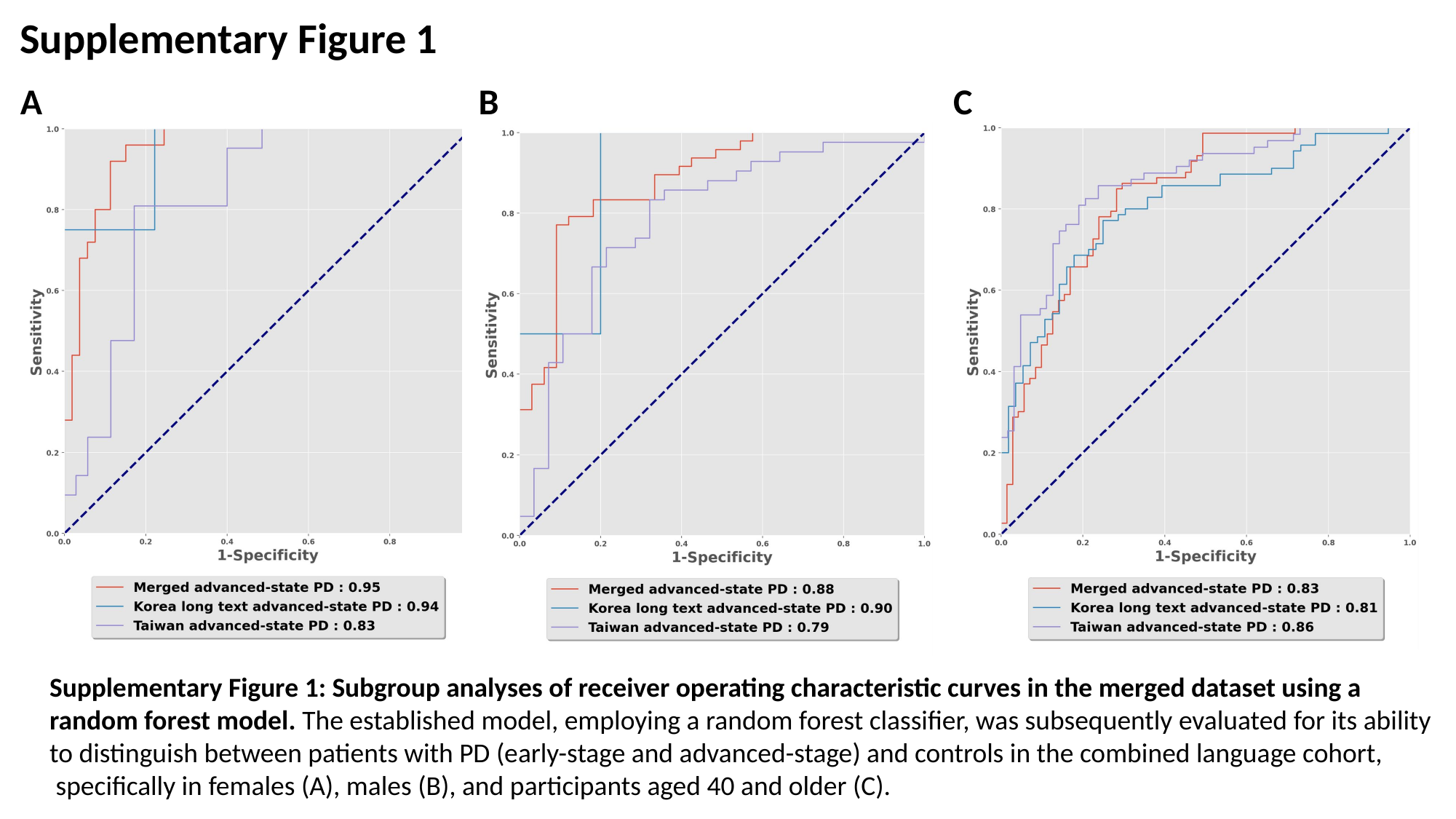

Supplementary Figure 1
B
C
A
Supplementary Figure 1: Subgroup analyses of receiver operating characteristic curves in the merged dataset using a
random forest model. The established model, employing a random forest classifier, was subsequently evaluated for its ability
to distinguish between patients with PD (early-stage and advanced-stage) and controls in the combined language cohort,
 specifically in females (A), males (B), and participants aged 40 and older (C).
