# Supplementary material for: A cross-language speech model for detection of Parkinson’s disease
Source: J Neural Transm (Vienna). 2024 Dec 30;132(4):579–90. doi: 10.1007/s00702-024-02874-z (PMC11909049; doi:10.1007/s00702-024-02874-z)
Supplement: Supplementary file 2 — Supplementary file2 (DOCX 2639 kb) [file 702_2024_2874_MOESM2_ESM.docx]

**Supplementary data:**

**Korean speech text**

Short Sentences:

1. 오늘 갈까요 아니면 내일 갈까요?
2. 엄마가 좋아요 아빠가 좋아요?
3. 우리나라의 가을은 참으로 아름답다.
4. 가을은 오곡백과 등 먹거리가 풍성하기 때문에 결실의 계절이라고도 한다.
5. 무엇보다도 산에 오를 땐 더욱 더 그 빼어난 아름다움이 느껴진다오늘 갈까요 아니면 내일 갈까요?
6. 엄마가 좋아요 아빠가 좋아요?
7. 우리나라의 가을은 참으로 아름답다.
8. 가을은 오곡백과 등 먹거리가 풍성하기 때문에 결실의 계절이라고도 한다.
9. 무엇보다도 산에 오를 땐 더욱 더 그 빼어난 아름다움이 느껴진다.

Long sentences:

- 1. 쓰다듬어진 듯한 완만함과, 깎아 놓은 듯한 뾰족함이 어우러진 산등성이를 따라 오르다 보면 절로 감탄을 금할 수가 없게 된다
  2. 붉은색, 푸른색, 노란색 등의 여러 가지 색깔들이 어우러져, 타는 듯한 감동을 주며 나아가 신비롭기까지 하다. 숲 속에 누워서 하늘을 바라보라
  3. 쌍쌍이 짝지어져 있는 듯한 흰 구름, 높고 파란 하늘을 쳐다보고 있노라면, 과연 예부터 가을을 천고마비의 계절이라 일컫는 이유를 알게 될 것만 같다.
  4. 새벽 동이 터 오르자 하늘로 열 기구가 서서히 떠오르기 시작했다. 구름처럼 가볍게 둥실둥실 떠다니는 열기구를 보자니 마치 예쁜 그림엽서 같았다.
  5. 전 세계적으로 기후 변화로 인해 평균 기온이 상승하고 있습니다. 이에 따라서 홍수, 가뭄, 해수면 상승 등 자연 재해가 자주 발생하고 있습니다.
  6. 거북이는 300살 이상을 살아서, 옛날부터 장수를 상징하는 동물로 여겨져왔다. 거북이는 다른 동물들에 비해 노화의 속도도 느린 것으로 밝혀졌다.
  7. 고양이는 또한 맹수의 상징이기도 했다. 고양이는 호랑이, 표범 등 잔혹한 맹수들과 함께 고양잇과에 속한다. 다만 그들 중 가장 작아서 인간이 길들일 수 있었을 뿐이다.

**Taiwanese speech dataset**

有一回，北風和太陽正在爭論誰的能耐大。爭來爭去，就是分不出個高低來。這會兒，來了個路人，他身上穿了件厚大衣。他們倆就說好了，誰能先叫這個路人把他的厚大衣脫下來，就算誰比較有本事。於是，北風就拚命地吹。怎料，他吹得越厲害，那個路人就把大衣包得越緊。最後，北風沒辦法，只好放棄。過了一陣子，太陽出來了。他火辣辣地曬了一下，那個路人就立刻把身上的厚大衣脫下來。於是，北風只好認輸了，他們倆之間還是太陽的能耐大。
