# Supplementary material for: A cross-language speech model for detection of Parkinson’s disease
Source: J Neural Transm (Vienna). 2024 Dec 30;132(4):579–90. doi: 10.1007/s00702-024-02874-z (PMC11909049; doi:10.1007/s00702-024-02874-z)
Supplement: Supplementary file 3 — Supplementary file3 (DOCX 20 kb) [file 702_2024_2874_MOESM3_ESM.docx]

**Supplementary Table 1 Speech Datasets Used in This Study.**

| **Language** | **Length of speech** | **Participant Group** | **Number of Participants** | **Character/Word Count (per task)** |
| --- | --- | --- | --- | --- |
| Korean | Short  (Sustained vowels, syllable repetition, sentence reading) | Healthy Controls | 125 | 1-40 characters |
|  |  | PD Patients (Early-stage) | 115 |  |
|  |  | PD Patients (advanced-stage) | 46 |  |
|  | Long  (text reading) | Healthy Controls | 125 | ≥40 characters |
|  |  | PD Patients (Early-stage) | 115 |  |
|  |  | PD Patients (advanced-stage) | 46 |  |
| Taiwanese Mandarin | Long | Healthy Controls | 174 | 500 characters |
|  | (text reading) | PD Patients (Early-stage) | 123 |  |
|  |  | PD Patients (Early-stage) | 63 |  |
